# Risk of uterine, ovarian and breast cancer following pelvic inflammatory disease: a nationwide population-based retrospective cohort study

**DOI:** 10.1186/s12885-016-2857-1

**Published:** 2016-11-03

**Authors:** Cheng-Che Shen, Li-Yu Hu, Albert C. Yang, Yung-Yen Chiang, Jeng-Hsiu Hung, Shih-Jen Tsai

**Affiliations:** 1 School of Medicine, National Yang-Ming University, Taipei, Taiwan; 2 Department of Psychiatry, Chiayi Branch, Taichung Veterans General Hospital, Chiayi, Taiwan; 3 Department of Information Management, National Chung-Cheng University, Chiayi, Taiwan; 4 Department of Psychiatry, Kaohsiung Veterans General Hospital, Kaohsiung, Taiwan; 5 Department of Psychiatry, Taipei Veterans General Hospital, Taipei, Taiwan; 6 Department of Dental Technology and Materials, Central Taiwan University of Science and Technology, Taichung, Taiwan; 7 Department of Obstetrics and Gynecology, Taipei Tzu Chi Hospital, Taipei, Taiwan; 8 School of Medicine, Tzu Chi University, Hualien, Taiwan

**Keywords:** Pelvic inflammatory disease, Uterine cancer, Ovarian cancer, Breast cancer, Retrospective cohort study

## Abstract

**Background:**

Pelvic inflammatory disease (PID) is characterized by infection and inflammation of the upper genital tract in women and is associated with health sequelae. We used a nationwide population-based retrospective cohort study to explore the relationship between PID and the subsequent development of gynecological cancers including ovarian, breast or uterine cancer.

**Methods:**

We identified subjects diagnosed with PID between January 1^st^, 2000 and December 31^st^, 2002 in the Taiwan National Health Insurance Research Database. A comparison cohort constructed for patients without PID were matched according to age and sex. All PID patients and control groups were observed until diagnosed with ovarian, breast or uterine cancer, or until death, withdrawal from the NHI system, or until December 31^st^, 2009.

**Results:**

The PID cohort consisted of 32,268 patients, and an equal number of matched controls without PID. The adjusted hazard ratio (HR) of ovarian, breast or uterine cancer in subjects with PID were: HR 1.326 (95 % confidence interval: 0.775–2.269), HR: 1.039 (95 % confidence interval: 0.862–1.252), and HR: 1.439 (95 % confidence interval: 0.853–2.426) respectively in comparison with controls during follow-up.

**Conclusions:**

This large nationwide population-based cohort study suggests that there is no increased risk for ovarian, breast or uterine cancer among women who have PID compared to a matching population.

**Electronic supplementary material:**

The online version of this article (doi:10.1186/s12885-016-2857-1) contains supplementary material, which is available to authorized users.

## Background

Pelvic inflammatory disease (PID) is a polymicrobial infection and inflammatory disorder of the upper female genital tract, including the uterus, fallopian tubes, and adjacent pelvic structures in women [1, 2]. It primarily affects young, sexually active women. Most women with PID can be treated successfully as outpatients with a course of antibiotics for at least two weeks [3].

Without adequate treatment, PID may lead to major sequelae, including ectopic pregnancy, pelvic pain, abscesses, and infertility. Since infertility and low parity are common complications of PID and are risk factors for ovarian cancer, a few studies have demonstrated that PID may increase the risk of ovarian cancer. A case-control study in Canada with 450 histologically verified primary epithelial ovarian cancer cases and 564 randomly selected population controls done by Risch and Howe, first reported that PID increased the risk of epithelial ovarian cancer [4]. This association has been interpreted in terms of inflammatory changes of the ovarian surface epithelium [4]. However, an Italian case–control study found no increased risk of ovarian cancer with a history of PID (OR 0.7, 95 % confidence interval, 0.4–1.3) [5]. Genetic differences between racial or ethnic groups, culture, and climate may partly explain these controversial results. Recently, Lin and colleagues used a large, nationwide health insurance database in Taiwan, demonstrating that the adjusted hazard ratio (HR) for ovarian cancer in patients with PID was 1.92 (95 % CI 1 · 27–2 · 92) during the 3-year follow-up period [6]. However, this study was limited by a short period between the PID and the diagnosis of ovarian cancer (1–3 years) [7]. Furthermore, patients with International Classification of Diseases, ninth revision, clinical modification (ICD-9-CM) code 616 (inflammatory disease of cervix, vagina, and vulva) could have been wrongly classified in PID cases in that study [8]. For these reasons, the first aim of this study is to explore whether PID increases the risk of developing ovarian cancer with the same database (Longitudinal Health Insurance Database 2005, LHID2005), but with a longer follow-up period excluding ICD-9-CM code 616 in PID cases. The second aim of this study to investigate whether women with PID raise the risk of developing uterine or breast cancer.

## Methods

### Data source

Instituted in 1995, the National Health Insurance (NHI) program is a mandatory health insurance program that offers comprehensive medical care coverage, including outpatient, inpatient, emergency, and traditional Chinese medicine, to all residents of Taiwan, with a coverage rate of up to 98 % [9]. The NHI research database (NHIRD) contains comprehensive information regarding clinical visits, prescription details and diagnostic codes based on ICD-9-CM. NHIRD is managed by the National Health Research Institutes, and confidentiality is maintained according to the directives of the Bureau of NHI. We used LHID2005 as our study data source. LHID2005 contains all the original claim data of 1,000,000 beneficiaries, randomly sampled from the year 2005 Registry for Beneficiaries (ID) of the NHIRD; everyone who was a beneficiary of the National Health Insurance Program within 2005 is in the population for random sampling. There are approximately 25.68 million individuals in this registry. All registration and claim data of the 1,000,000 individuals collected by the National Health Insurance Program constitute the LHID2005. There were no significant differences in gender distribution, age distribution, or average insured payroll-related amount between the patients in the LHID2005 and those in the original NHIRD (http://nhird.nhri.org.tw/en/index.htm). Recently, using LHID2005, we have demonstrated an increased risk of uterine but not ovarian and breast cancer in women with polycystic ovary syndrome [10].

### Study population

Using data extracted from the LHID2005, we conducted a retrospective cohort study of patients who were newly diagnosed with PID by an obstetrician-gynecologist between January 1^st^, 2000 and December 31^st^, 2002. To ensure diagnostic validity and patient homogeneity, we selected only patients who had at least two consensus PID diagnoses for the study group. We excluded patients who were diagnosed with PID between January 1^st^, 1996, and December 31^st^, 1999. We also excluded patients who were diagnosed with malignancies (ICD-9-CM codes: 140-208) before they were diagnosed with PID. For every PID patient included in the final cohort, one age- and sex-matched control without PID, and any malignancy was randomly selected from LHID 2005 in the same time period as PID patient. Random assignment procedures were performed by SAS statistical software and were based on random numbers generated from the uniform distribution. All PID patients and controls were observed until diagnosed with breast cancer (ICD-9-CM code: 174-175), ovarian cancer (ICD-9-CM codes: 183), uterine cancer (ICD-9-CM codes: 179, 181 and 182), or until death, withdrawal from the NHI system, or until December 31^st^, 2009. Our main dependent variable was the occurrence of breast cancer, ovarian cancer, or uterine cancer, as reported in the Registry for Catastrophic Illness. For a diagnosis of cancer to be reported in the Registry, histological confirmation is required. Common co-morbidities including hypertension, diabetes mellitus, dyslipidemia, congestive heart failure, chronic pulmonary diseases, coronary artery diseases, and cerebrovascular diseases were also compared between PID and controls. The study design and the criteria had been used in similar studies [10–12].

### Statistical analysis

The incidences of newly diagnosed breast cancer, ovarian cancer, or uterine cancer in PID patients and controls were calculated, and independent *t* tests and chi-squared tests were used to examine differences in demographic characteristics between the PID patients and controls. A Cox proportional-hazards regression model was constructed to calculate the hazard ratio (HR) of breast cancer, ovarian cancer, or uterine cancer of the PID and control cohorts, respectively. Control variables, such as age, urbanization, monthly income, common co-morbidities including hypertension, diabetes mellitus, dyslipidemia, congestive heart failure, chronic pulmonary diseases, coronary artery diseases, and cerebrovascular diseases were included as covariates in the multivariate model to calculate adjusted HR. SAS statistical software for Windows, version 9.3 (SAS Institute, Cary, NC, USA), was used for data extraction, computation, data linkage, processing, and sampling. All other statistical analyses were performed using SPSS statistical software for Windows, version 20 (IBM, Armonk, NY, USA). Results for comparisons with a *P* value of less than .05 were considered as a statistically significant relationship.

### Case control study design

For further confirmation of our study results, we also designed a case control study. All subjects who were aged 20 and older and were newly diagnosed with uterine, ovarian or breast cancer, respectively in 2009 were included as case group. The controls were matched to cases by age, sex, and index date with a ratio of 1:4. Diagnosis of PID before index events was recorded during period of 2000-2009. The percentage of PID diagnosis in patients with uterine, ovarian, breast cancer and three control cohorts were calculated respectively. Odds ratios and 95 % confidence intervals were also estimated by using multiple logistic regression.

## Results

Our study included 32,268 PID patients and 32,268 controls without PID. Comparisons of demographic and clinical variables between PID patients and controls are presented in Table 1. Median age at enrollment was 34.48 years (inter-quartile range [IQR], 27.46–42.06 years), with a median follow-up period of 8.84 years (IQR, 8.04–9.51 years) for both PID and controls. Co-morbidities, including hypertension, diabetes mellitus, dyslipidemia, congestive heart failure, cerebrovascular disease and chronic pulmonary disease were more common in PID patients than controls. During this study period, 34 ovarian cancers, 228 breast cancers, and 35 uterine cancers were observed in PID group and 24 ovarian cancers, 222 breast cancers, and 27 uterine cancers were observed in control group.

**Table 1:** Characteristics of patients with pelvic inflammatory disease and comparison subjects

	PID (%)	Control (%)	*P* values
No.	32,268	32,268	
Age (years) ^a^	34.48 (27.46 – 42.06)	34.48 (27.47 – 42.06)	0.972
Distribution of age			>0.999
20 – 39	22,221 (68.86)	22,221 (68.86)	
40 – 59	9299 (28.82)	9299 (28.82)	
> 60	748 (2.32)	748 (2.32)	
Comorbidities			
Hypertension	1985 (6.15)	1640 (5.08)	<0.001^b^
Diabetes mellitus	1452 (4.50)	1106 (3.43)	< 0.001^b^
Dyslipidemia	1816 (5.63)	1266 (3.92)	< 0.001^b^
Coronary artery disease	51 (0.16)	37 (0.11)	0.137
Congestive heart failure	181 (0.56)	132 (0.41)	0.006^b^
Cerebrovascular disease	437 (1.35)	348 (1.08)	0.002^b^
Chronic pulmonary disease	1684 (5.22)	1230 (3.81)	< 0.001^b^
Degree of urbanization			< 0.001^b^
Urban	20,634 (63.95)	21,167 (65.60)	
Suburban	8,998(27.89)	8,632 (26.75)	
Rural	2,159 (6.69)	1,971 (6.11)	
Income group			< 0.001^b^
Low income	13,021 (40.35)	13,978 (43.32)	
Median income	15,719 (48.71)	13,016 (40.34)	
High income	3,528 (10.93)	5,274 (16.34)	
Follow-up, years ^a^	8.84 (8.04 – 9.51)	8.84 (8.04 – 9.51)	>0.999
Newly diagnosed cancers, N (%)			
Ovarian cancer	34 (0.11)	24 (0.07)	0.192
Breast cancer	228 (0.71)	222 (0.69)	0.777
Uterine cancer	35 (0.11)	27 (0.08)	0.313
Age of diagnosis of cancer (years) ^a^			
Ovarian cancer	45.50 (40.75 – 59.00)	46.50 (41.25 – 51.75)	0.183
Breast cancer	46.00 (40.00 – 52.00)	45.00 (41.00 – 50.00)	0.397
Uterine cancer	47.00 (40.00 – 56.00)	52.00 (45.00 – 55.00)	0.222

*PID* pelvic inflammatory disease

^a^ Median (interquartile range)

^b^Statistical significance

### PID on risks of ovarian cancer, breast cancer, and uterine cancer

After adjusting for age, co-morbidities, urbanization, and monthly income, the adjusted HR of ovarian, breast and uterine cancer in subjects with PID were 1.33 (95 % confidence interval [CI] 0.78–2.27), 1.04 (95 % CI 0.86–1.25), 1.44 (95 % CI 0.85–2.43) respectively in comparison with controls during follow-up (Table 2).

**Table 2:** Hazard ratios of developing cancer between patients with pelvic inflammatory disease and comparison subjects

	Crude HR (95 % CI)	Adjusted HR (95 % CI)^a^
Ovarian cancer	1.42 (0.84 – 2.39)	1.33 (0.78 – 2.27)
Breast cancer	1.03 (0.85 – 1.24)	1.04 (0.86 – 1.25)
Uterine cancer	1.35 (0.81 – 2.24)	1.44 (0.853 – 2.43)

*HR* hazard ratio, *CI* confidence interval

^a^Adjusted for age, hypertension, diabetes mellitus, dyslipidemia, coronary artery disease, congestive heart failure, cerebrovascular disease, chronic pulmonary disease, urbanization and income

### Results of case control study design

The percentage of PID history in patients with ovarian, breast and uterine cancer, were 29.09 %, 19.38 %, 28.57 %, respectively (Tables 3, 4 and 5). The Odds ratios of ovarian, breast and uterine cancer in subjects with PID history at index date were 1.40 (95 % CI 0.72–2.70), 1.12 (95 % CI 0.84–1.51), 1.64 (95 % CI 0.90–2.97) respectively. The results are consistent with our cohort study designs which showed that there is no increased risk for ovarian, breast or uterine cancer among women who have PID compared to a matching population.

**Table 3:** Odds ratio of history of pelvic inflammatory disease on ovarian cancer

	Ovarian cancer group (N = 55)	Control group (N = 220)	OR = 1.40 (95 % CI 0.72–2.70) *P* = 0.32
PID (%)	16 (29.09)	50 (22.73)	

*OR* odds ratio, *CI* confidence interval, *PID* pelvic inflammatory disease

**Table 4:** Odds ratio of history of pelvic inflammatory disease on breast cancer

	Breast cancer group (N = 356)	Control group (N = 1424)	OR = 1.12 (95% CI 0.84–1.51) *P* = 0.44
PID (%)	69 (19.38)	251 (17.63)	

*OR* odds ratio, *CI* confidence interval, *PID* pelvic inflammatory disease

**Table 5:** Odds ratio of history of pelvic inflammatory disease on uterine cancer

	Uterine cancer group (N = 70)	Control group (N = 280)	OR = 1.64 (95 % CI 0.90–2.97) *P* = 0.11
PID (%)	20 (28.57)	55 (19.64)	

*OR* odds ratio, *CI* confidence interval, *PID* pelvic inflammatory disease

## Discussion

In a nationwide population-based study of ovarian cancer among PID patients in Taiwan, it was found that patients with PID had an increased risk of ovarian cancer than patients without PID [6]. However, in our nationwide population-based study in Taiwan with the same database, no significantly increased risk of subsequent ovarian cancer was observed after a diagnosis of PID. The discrepancy between such study and our study may have come from differences in study periods (3 years versus 10 years), physician specialties for PID diagnosis (obstetrician-gynecologist and other specialties versus obstetrician–gynecologist), differences in diagnosis restrictions for ovarian cancer (only patients with catastrophic illness certificate in our study) and PID ICD-9-CM coding (ICD-9-CM codes 614-616 versus ICD-9-CM codes: 614-615). In the cohort studied by Lin and colleagues, at least two episodes of PID in relation to diagnosis of ovarian cancer had to occur within 1–3 years [6]. In such a short period, a causal relation between PID and ovarian cancer seems less likely, and the inverse relationship between ovarian cancer risk factor and PID cannot be excluded [7]. Furthermore, both ICD-9-CM codes 614 and 615 refer to inflammatory disease of the ovary, fallopian tube, pelvic cellular tissue, peritoneum, and uterus, except cervix, respectively, which fit the diagnosis of PID. However, PID cases in Lin and colleagues’ study also included ICD- 9-CM code 616, which refers to the inflammatory disease of cervix, vagina, and vulva. These infections affect only the lower genital tract which is unrelated to or has not yet progressed to PID. Thus, it would be inappropriate to include diseases pertinent to code 616 in this survey [8]. In addition, our main dependent variables, the occurrence of breast cancer, ovarian cancer, or uterine cancer, are collected from the Registry for Catastrophic Illness and histological confirmation is required for a diagnosis of cancer to be reported in the Registry, Therefore, the diagnosis of cancer in our study is more precise.

Furthermore, our analysis showed that hypertension, dyslipidemia and diabetes were more prevalent in women with PID than in women without PID (Table 1). Medical conditions associated with metabolic syndrome such as hypertension, dyslipidemia and diabetes may alter immune response and increase the risk of PID in women [13, 14].

Ovarian, uterine and breast cancers are associated with several risk factors, such as low parity and infertility [15], which are common complications of PID [16, 17]. In addition, inflammatory response triggered by bacterial or viral infection is a major factor for human carcinogenesis [18, 19]. In addition of ovarian cancer, earlier reports have suggested PID diagnosis is a risk factor for cervical intra-epithelial neoplasia [20], cervical cancer [21] and colorectal cancer [22]. In this study, we investigated the hypothesis that PID may increase the risk of uterine and breast cancers using a national health insurance database. Our study suggests that there are no increased risks for both cancers among people who have PID compared to a matching population.

Based on the relatively small number of patients diagnosed with cancer in our study, we collected annual number and incidence of ovarian, breast and uterine cancer of Taiwan from Health Promotion Administration, Ministry of Health and Welfare of Taiwan (https://cris.hpa.gov.tw/) and compared above figures with our study. The results showed that incidence rate of ovarian, breast or uterine cancer was similar between general population of Taiwan and subjects of our study (Additional file 1: Table S1). Hence, we think the results of our study were representative and valid.

The strength of our study is the use of a population-based data set with an enrollment of a large number of subjects, enabling us to trace the subsequent risk of gynecologic cancers in PID subjects and controls. Furthermore, diagnosis of cancer in our study must be confirmed by histological confirmation. In addition, our study design included an unbiased participant selection process. Because participation in NHI is mandatory and all residents of Taiwan can access health care with low copayments, referral biases were low with high follow-up compliance.

Certain limitations to our findings should, however, be considered: First, LHID2005 did not contain some important information regarding the studied subjects. Some of the patient information which may influence the risk of gynecologic cancers, such as smoking, alcohol consumption, parity, infertility, age at menarche, and family history of gynecologic cancers, were not available for analysis [15]. Thus, we were unable to control these potentially confounding factors. Second, diagnosis of PID can only be confirmed with a positive bacteriological test for lower genital tract infection such as chlamydia or gonorrhea [23]. PID diagnosis was entirely determined using the ICD codes from the National Health Insurance claim database, and there may be concerns regarding the diagnostic accuracy of the database. Third, the follow-up duration in this study (median follow-up time 8.84 years) may have been insufficient for detecting the carcinogenesis of certain types of cancer. Most ovarian, uterine, and breast cancer occur after age 50 and the short follow-up time in this study may underestimate any possible association. Thus, future studies with longer follow-up periods are required to determine the long-term risk of cancer among PID patients. Forth, our study is based on Taiwan National Health Insurance research database and it’s difficult to apply the results of our study to other regions with complete different population composition, culture, or climate due to limited geographical coverage. Finally, prevalence of PID diagnoses were identified using the ICD-9 codes from the database may be underestimated as only subjects seeking medical evaluation can be identified, leading to a likely underestimation of the association between PID and gynecologic cancers.

## Conclusions

In summary, our study did not support the idea that the risk of ovarian cancer is higher in PID patients as shown in an earlier report with same nationally representative cohort database. We also demonstrated that PID diagnosis is not a risk factor for breast or uterine cancers.

## Additional file

Additional file 1: Table S1. Number and incidence rate (IR, cancer event per 100000 person years) of ovarian, breast or uterine cancer in general population of Taiwan and PID subjects between 2000 and 2009. (DOC 49 kb)
