# Supplementary material for: Risk of uterine, ovarian and breast cancer following pelvic inflammatory disease: a nationwide population-based retrospective cohort study
Source: BMC Cancer. 2016 Nov 3;16:839. doi: 10.1186/s12885-016-2857-1 (PMC5094045; doi:10.1186/s12885-016-2857-1)
Supplement: Additional file 1: Table S1. — Number and incidence rate (IR, cancer event per 100000 person years) of ovarian, breast or uterine cancer in general population of Taiwan and PID subjects between 2000 and 2009. (DOC 49 kb) [file 12885_2016_2857_MOESM1_ESM.doc]

**Table S1.** Number and incidence rate (IR, cancer event per 100000 person years) of ovarian, breast or uterine cancer in general population of Taiwan and PID subjects between 2000 and 2009

|  | Ovarian cancer | | | | | | |
| --- | --- | --- | --- | --- | --- | --- | --- |
|  | General population | | |  | PID | | |
|  | Number |  | IR |  | Number |  | IR |
| Distribution of age |  |  |  |  |  |  |  |
| 20–39 | 1712 |  | 4.63 |  | 7 |  | 4.008406 |
| 40–59 | 4862 |  | 15.55 |  | 20 |  | 14.94981 |
| >60 | 2633 |  | 17.50 |  | 7 |  | 53.30085 |
| Total | 9207 |  | 11.05 |  | 34 |  | 10.57388 |

|  | Breast cancer | | | | | | |
| --- | --- | --- | --- | --- | --- | --- | --- |
|  | General population | | |  | PID | | |
|  | Number |  | IR |  | Number |  | IR |
| Distribution of age |  |  |  |  |  |  |  |
| 20–39 | 8813 |  | 23.83 |  | 49 |  | 28.06 |
| 40–59 | 40410 |  | 129.24 |  | 164 |  | 122.59 |
| >60 | 16094 |  | 106.94 |  | 15 |  | 114.22 |
| Total | 65317 |  | 78.42 |  | 228 |  | 70.91 |

|  | Uterine cancer | | | | | | |
| --- | --- | --- | --- | --- | --- | --- | --- |
|  | General population | | |  | PID | | |
|  | Number |  | IR |  | Number |  | IR |
| Distribution of age |  |  |  |  |  |  |  |
| 20–39 | 1057 |  | 2.86 |  | 8 |  | 4.58 |
| 40–59 | 6580 |  | 21.04 |  | 24 |  | 17.94 |
| >60 | 2583 |  | 17.16 |  | 3 |  | 22.84 |
| Total | 10220 |  | 12.27 |  | 35 |  | 10.88 |
